# Supplementary material for: Deregulated PP1α phosphatase activity towards MAPK activation is antagonized by a tumor suppressive failsafe mechanism
Source: Nat Commun. 2018 Jan 15;9:159. doi: 10.1038/s41467-017-02272-y (PMC5768788; doi:10.1038/s41467-017-02272-y)
Supplement: Supplementary file 1 — Supplementary Information [file 41467_2017_2272_MOESM1_ESM.pdf]

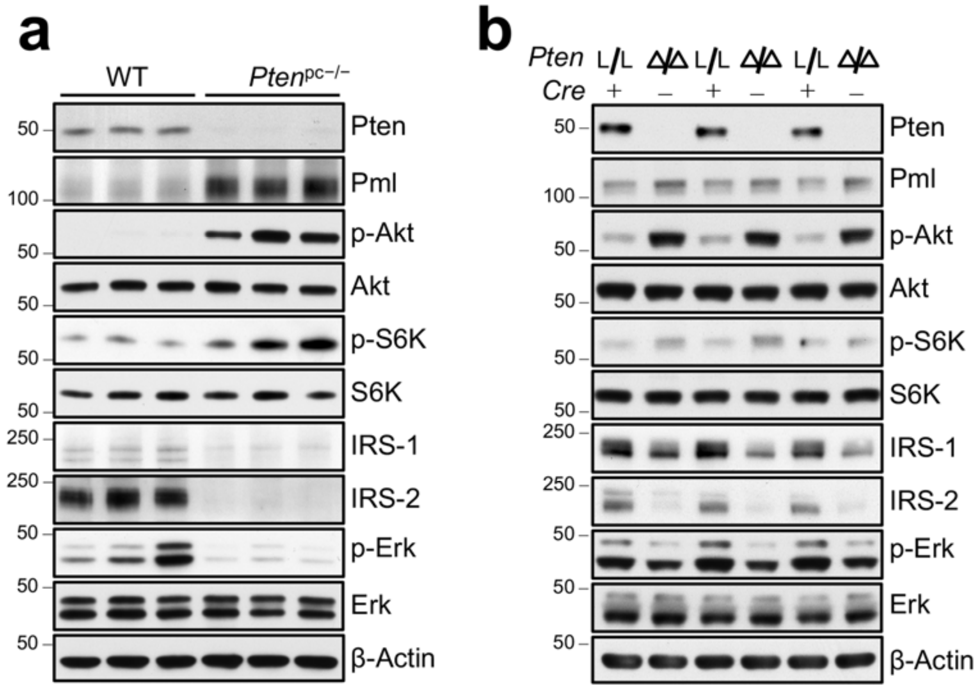

Supplementary Figure 1 *PTEN* loss leads to feedback inhibition of ERK-MAPK signaling. (**a**,**b**) IB analysis of dorsal-later prostate tissues from wild type and prostate epithelium-specific *Pten* inactivation (*Pten*$^{pc-/-}$) mice at 12 weeks of age (**a**), lysates from primary *Pten*$^{lox/lox}$ MEFs transduced with control or *Cre* retrovirus at 72 hrs after selection (**b**).

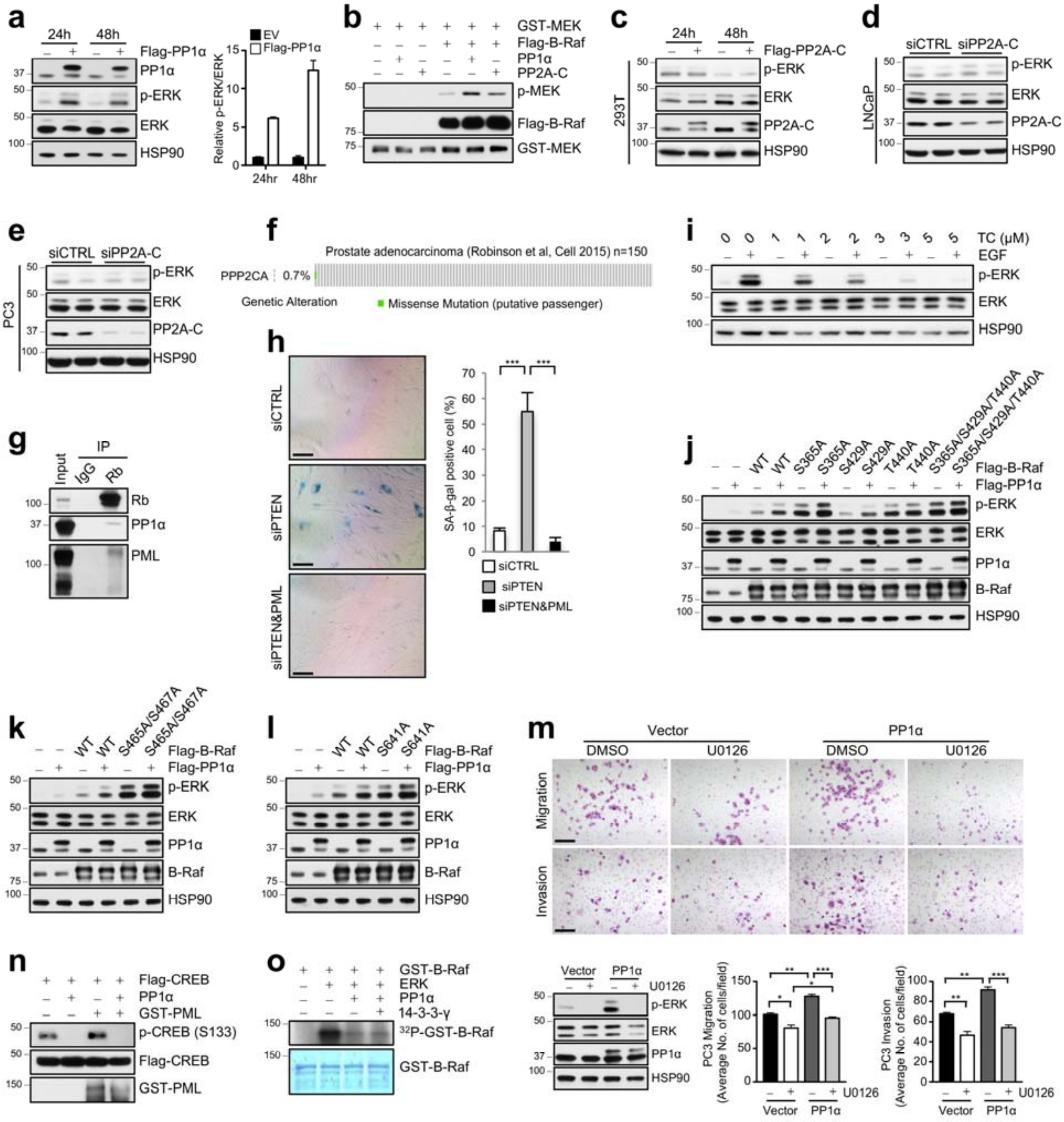

Supplementary Figure 2 PP1α mediates *PML*-loss induced MAPK activation and promotes CaP cell invasiveness through activation of MAPK signaling. (**a**) Immunoblot (IB) analysis of lysates from LNCaP cells transfected with EV or Flag-PP1α for the indicated time periods. Quantification of p-ERK/ERK levels was carried out with Image J software. Numbers indicate

the relative ratios to controls for phosphoprotein/total protein. Data shown are mean ± s.e.m. of three independent experiments. (**b**) *In vitro* kinase assays showing that both PP2A-C and PP1α promoted B-Raf kinase activity towards phosphorylating GST-MEK1. (**c-e**) IB analysis of lysates from 293T cells transfected with empty vector or PP2A-C for the indicated times (**c**), lysates from LNCaP (**d**) or PC3 cells (**e**) transfected with control or PP2A-C siRNA for 48 hrs. (**f**) Genetic alterations of *PPP2CA* in the Robinson *et al.* dataset[9]. The gene alteration percentages are shown. It should be noted that neither genomic amplification nor deletion was observed for PPP2CA in this dataset of 150 samples from mCRPC patients. (**g**) Endogenous co-immunoprecipitation of Rb□with PP1α and PML in□PC3 cells. Input is 10% of total cell extracts used for immunoprecipitation. (**h**) Cytochemical staining and quantification of senescence-associated β-galactosidase (SA-β-gal) activity in WI-38 cells transfected with control, PTEN siRNA or PTEN plus PML siRNA at 5 days post-transfection. Data shown are mean ± s.d. of three independent experiments. ***P<0.001 by unpaired two-tailed *t*-test. Scale bar, 100μm. (**i**) IB analysis of lysates from serum-starved PC3 cells pretreated with tautomycin at the indicated concentration for 3 hrs, followed by stimulation with 10ng/ml EGF for 5 min. (**j-l**) IB analysis of lysates from 293T cells transfected with the indicated WT or mutant Flag-B-Raf constructs plus EV or Flag-PP1α for 24 hrs. (**m**) Representative images and quantitation of migrated and invaded PC3 cells in the migration and invasion assay. PC3 stable cells were subjected to migration or invasion assay (24 hrs) (n=3 per group, 4 fields per insert) in the absence or presence of 20μM U0126. Western blotting confirmed the expression of phosphor-ERK and PP1α. Data shown are mean ± s.e.m. of three independent experiments. *P<0.05, **P<0.01, ***P<0.001 by unpaired two-tailed *t*-test. Scale bar, 100μm. (**n**) *In vitro* phosphatase assays showing that PML did not affect PP1α phosphatase activity towards dephosphorylating

Flag-CREB. (**o**) *In vitro* kinase and phosphatase assays showing that 14-3-3γ did not affect PP1α phosphatase activity towards dephosphorylating GST-B-Raf.

Figure 2a

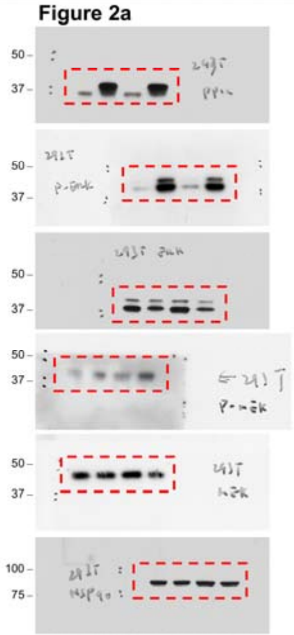

Figure 2b

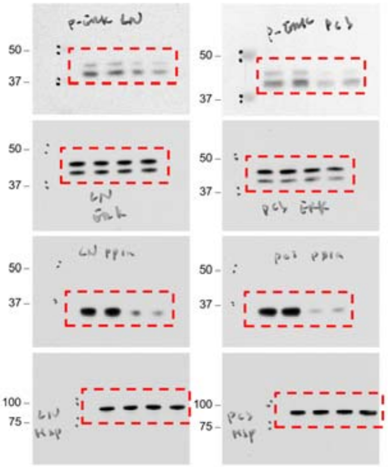

Figure 2d

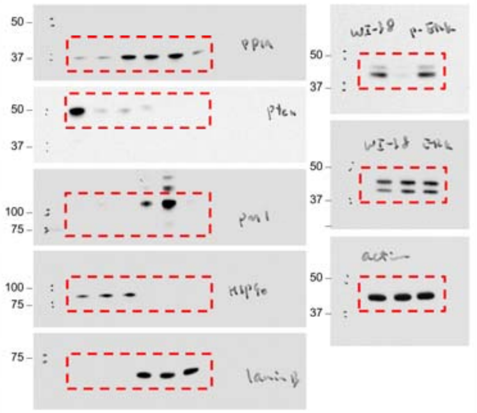

Figure 2e

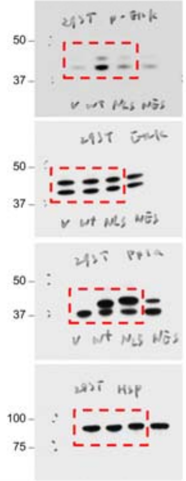

Figure 2f

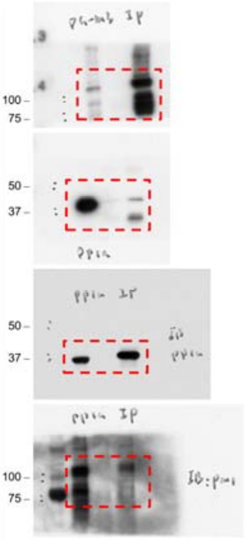

Figure 2g

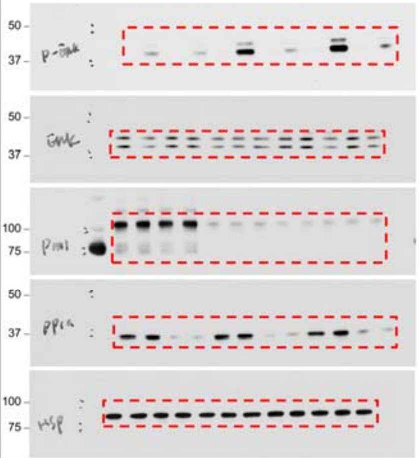

Figure 2h

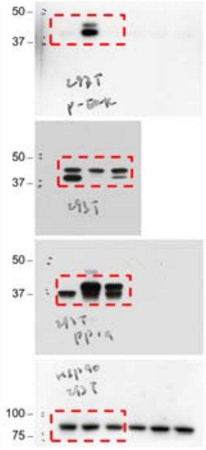

Figure 3a

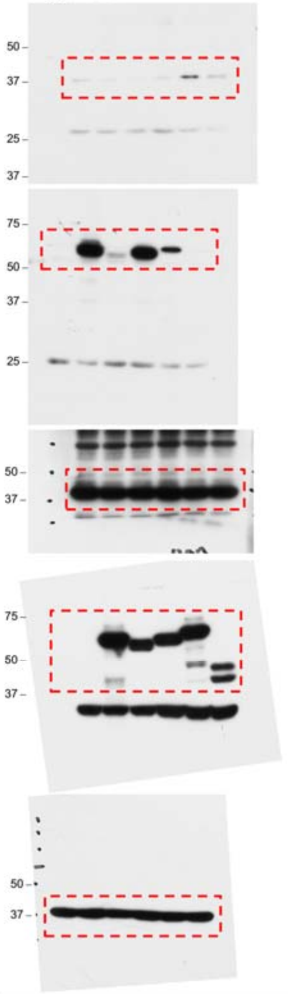

Figure 3b

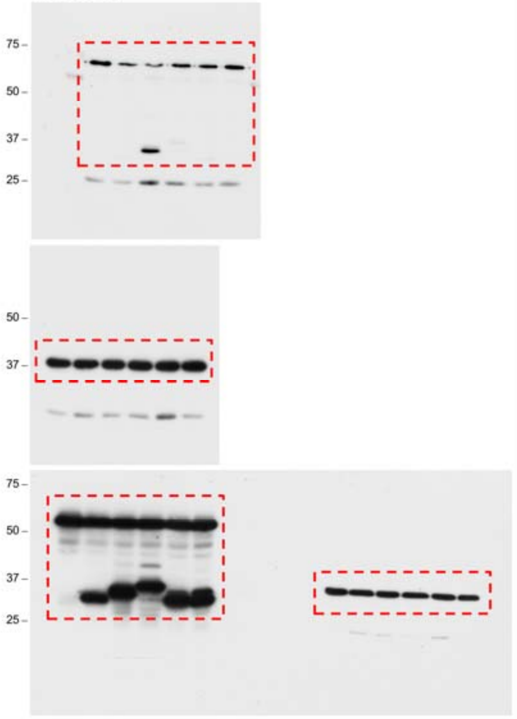

Figure 3c

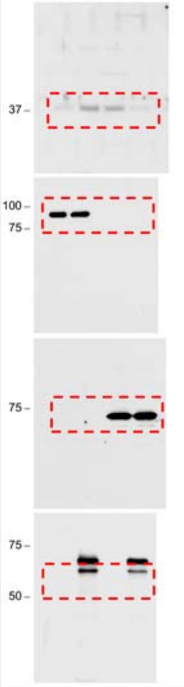

Figure 3e

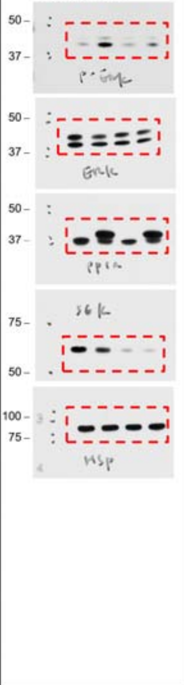

Figure 3g

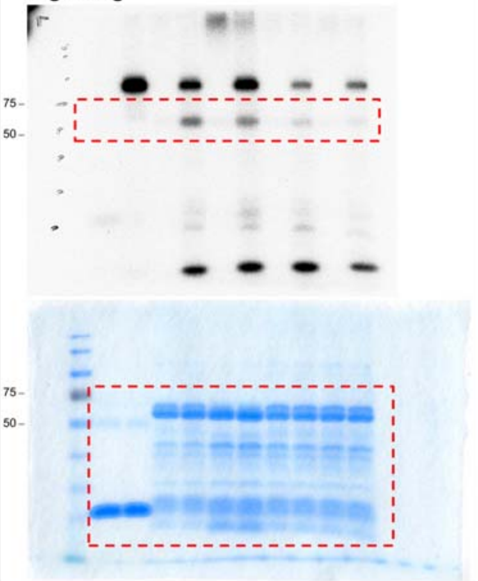

Figure 3h

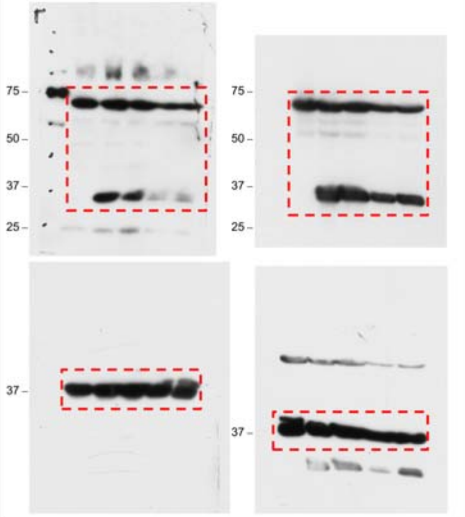

Figure 3i

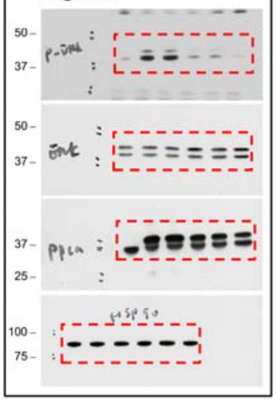

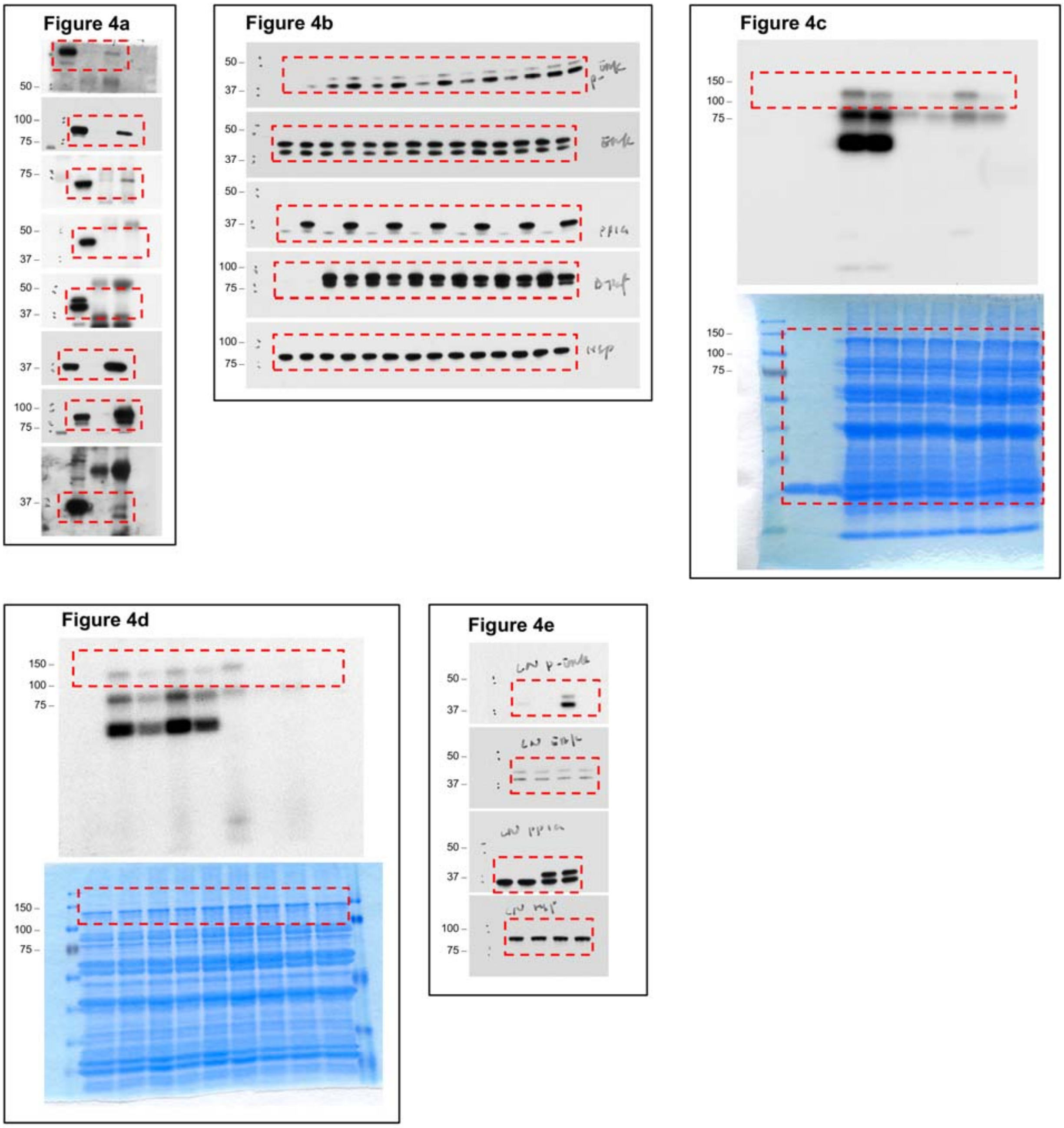

Supplementary Figure 3 Uncropped scans for Western blot data in main figures.

Supplementary Table 1. Sequences of siRNAs used in this study

**Names**	**Sequences of siRNAs**
h-PML-siRNA1	5’-GCAACCAGUCGGUGCGUGA-3’
h-PML-siRNA2	5’-CCGACUUCUGGUGCUUUGA-3’
h-PTEN-siRNA-SMARTpool	5’-GUGAAGAUCUUGACCAAUG-3’
	5’-GAUCAGCAUACACAAAUUA-3’
	5’-GGCGCUAUGUGUAUUAUUA-3’
	5’-GUAUAGAGCGUGCAGAUAA-3’
h-PPP1CA-siRNA-1	5’-CCGCAUCUAUGGUUUCUAC-3’
h-PPP1CA-siRNA-2	5’-CAUCUAUGGUUUCUACGAU-3’
h-PPP2CA-siRNA-1	5’-CUGGUUACACCUUUGGGCA-3’
h-PPP2CA-siRNA-2	5’-UAACCAAGCUGCAAUCAUG-3’
h-S6K1-siRNA-1	5’-GGAAUAUUUAUGGAAGACA-3’
h-S6K1-siRNA-2	5’-GAUAGUAAGAAAUGCUAAA-3’
siGENOME Non-Targeting siRNA #2	5’-UAAGGCUAUGAAGAGAUAC-3’
